# Detecting groundwater level changes related to the 2016 Kumamoto Earthquake

**DOI:** 10.1038/s41598-023-50133-0

**Published:** 2023-12-21

**Authors:** Shun Yamamoto, Katsuaki Koike, Yosuke Alexandre Yamashiki, Jun Shimada

**Affiliations:** 1 Graduate School of Advanced Integrated Studies in Human Survivability, Kyoto University, Kyoto, Japan; 2 Graduate School of Engineering, Kyoto University, Kyoto, Japan; 3 Graduate School of Science and Technology, Kumamoto University, Kumamoto, Japan

**Keywords:** Seismology, Hydrogeology

## Abstract

This study presented the first attempt to detect precursory changes in groundwater level before the 2016 Kumamoto Earthquake. This detection was achieved by accurately determining the relationship between long-term groundwater level fluctuation and crustal deformation over 16 years through analysis of groundwater level time-series data acquired at 17 sites within the study area. Here, we show that the observed groundwater levels were lower than the modelled levels in aquifers composed of porous strata (Togawa lava and part of the pre-Aso volcanic rocks), and that there were larger differences until 2014, which diminished until the occurrence of the Kumamoto Earthquake. The initial reduction in the modelled groundwater level and the latter recovery were most likely caused by crustal strain relaxation associated with the large 2011 earthquake off the Pacific coast of Tohoku (M_w_ 9.0) and the strain accumulation prior to the 2016 Kumamoto Earthquake.

## Introduction

The main sources of groundwater regeneration are rainfall and river water, and changes in these sources affect groundwater level. However, groundwater level is also affected by atmospheric pressure, earth tides, and earthquakes^1^. Earthquake-related change in groundwater level occurs not only concurrently with an earthquake event, but also before and after an earthquake in association with variation in crustal deformation and strain caused by the regional stress field. Understanding the correlation between groundwater level fluctuations and earthquakes can provide new insight into the spatiotemporal variability of hydrologic properties and processes^2^. In Japan, traditional analysis is based on multivariate linear regression of groundwater level data that adopts other factors as explanatory variables, which can distinguish changes in groundwater level caused explicitly by an earthquake, from those manifested prior to the occurrence of an earthquake^3^. The frequency of groundwater level fluctuations was also characterized using a Hilbert-Huang Transform analysis. This signal processing method is effective for nonlinear and non-stationary data and helps to identify abnormal rises and falls in groundwater levels. The 1999 Chi-Chi earthquake (M_w_ 7.7) in Taiwan showed a significant correlation in abnormal changes with the surface displacement near the epicenter. This finding highlights the capability of the Hilbert-Huang transformation as a potential method for detecting precursors of earthquakes^4^. In mainland China, integrating diverse observational data such as foreshocks, geophysical precursors, and local reports of macro-anomalies was demonstrated to be vital for earthquake prediction by a partial success in the prediction of the 1975 Haicheng earthquake^5,6^.

In the Kumamoto area of southwestern Japan, with a population of approximately 740,000, all water for domestic, agricultural, and industrial use is supplied by groundwater^7^. Accordingly, groundwater level observations are conducted at many wells to conserve and manage groundwater resources. This region experienced the 2016 Kumamoto Earthquake that comprised the foreshock (M_w_ 6.2) at 21:26 (all times Japan standard time) on 14 April 2016 and the subsequent mainshock (M_w_ 7.0) at 01:25 on 16 April 2016. Relationships between this earthquake and groundwater properties have been studied from various aspects, including an increase in aquifer permeability using water stable isotope ratios^8^, temporal changes in the chemical composition of the water^9,10^, changes in river levels before and after the earthquake^11^, and observed helium isotope fluctuations^12^. However, most of these studies primarily concentrated on the groundwater changes after the earthquake. Precursory changes must have occurred before large earthquakes, although the changes may be much smaller than the later changes and difficult to be detected. Only a few studies have attempted to characterize the fluctuations in groundwater levels before the 2016 Kumamoto Earthquake. One such example involved the application of a tank model to reproduce water level changes from 2 years prior to 3 years after the occurrence of an earthquake using precipitation and groundwater level fluctuation data^13^. Another example, which used precipitation and pressure time series data^14^ to conduct a qualitative comparison of groundwater level changes for the 10 years prior to the Kumamoto Earthquake, concluded that no evident change in water level occurred prior to the earthquake. Long-term groundwater level fluctuations before earthquakes have not yet been studied comprehensively through time-series analysis, and therefore the precursory changes in water level, controlling factors, and differences in terms of location remain unknown.

Based on the above, this study characterised the long-term changes in groundwater level and detected the precursory features prior to the 2016 Kumamoto Earthquake. This was achieved using a multivariate linear regression model that adopted water level as the objective variable and took precipitation, atmospheric pressure, and the earth tide as explanatory variables. Differences between the model-derived and observed groundwater levels were correlated with aquifer lithology and global navigation satellite system (GNSS) crustal strain data. Both the large 2011 earthquake off the Pacific coast of Tohoku on 11 March 2011 (M_w_ 9.0) and the 2016 Kumamoto Earthquake might have influenced crustal deformation. The validity of the model and the interpretation of the main controlling factors on the spatiotemporal changes in groundwater levels are discussed in relation to reference cases of recent smaller earthquakes that occurred on 3 January 2019 (M_w_ 4.9)^15^ and on 26 June 2022 (M_j_ 4.7)^16^.

**Figure 1:**
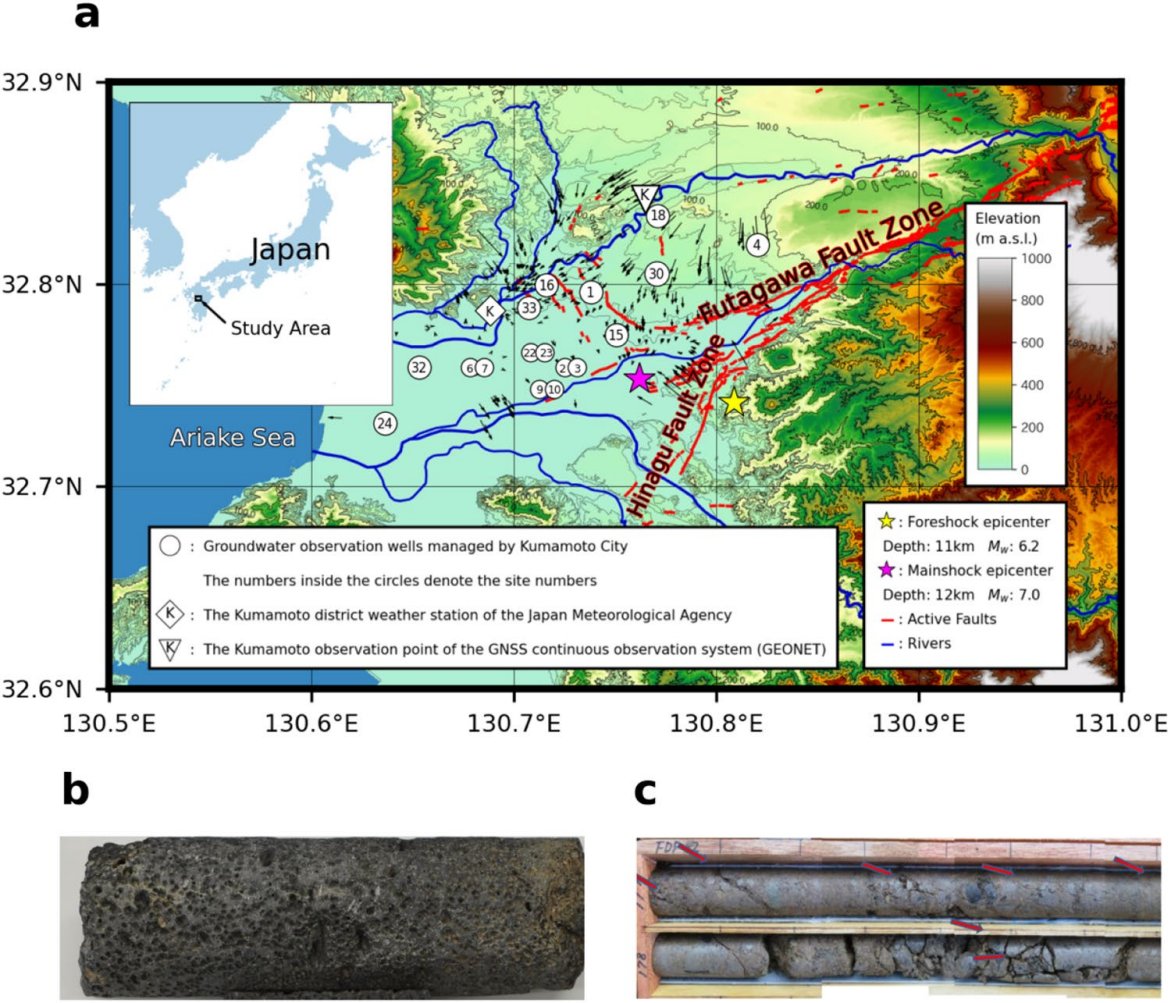
Location of the 2016 Kumamoto Earthquake, distribution of related faults, and location of groundwater observation wells. (**a**) Location and topography of the Kumamoto area in southwestern Japan, overlain with active fault traces from the active fault database^19^; position of the epicenter of the foreshock and the mainshock of the 2016 Kumamoto Earthquake; location of the 13 sites for the 17 groundwater observation wells managed by Kumamoto City; location of the Kumamoto District Weather Station of the Japan Meteorological Agency; location of Kumamoto station of the GNSS Continuous Observation System (GEONET) of the Geospatial Information Authority of Japan^20^; surface geological map of the Kumamoto area and regional groundwater flow system produced through a trend surface analysis of groundwater level data^7^. (**b**) Photo of porous portion of the Togawa lava from core samples. c, Photo of fractured portions of the upper pre-Aso volcanic rocks from borehole cores^21^.

A conspicuous feature of the 2016 Kumamoto Earthquake was the successive occurrence of the foreshock and the mainshock within a short period with epicenters (32° 44.5ʹ N, 130° 48.5ʹ E) and (32° 45.2ʹ N, 130° 45.7ʹ E) located closely, at depths of approximately 4.5 km (Fig. 1a). Their hypocentre depths were also nearly the same, i.e., 11 and 12 km, respectively. Both earthquakes were caused by movements of the Futagawa and Hinagu faults^17^, which are the major active faults within the study area (Fig. 1a). In addition to the substantial and extensive damage caused to buildings and infrastructure, and the widespread occurrence of landslides, the earthquakes affected the groundwater system and changed the amount of spring water and groundwater discharge^18^.

The Kumamoto area is topographically separated into two main zones: an alluvial lowland formed by the Shirakawa and Midorikawa rivers in the west, and a diluvial terrace that extends to the outer rim of Mt. Aso in the east (Fig. 1a). From approximately 270 to 90 ka, Mt. Aso had four large eruptions that produced surrounding pyroclastic flow deposits, namely, Aso-1, 2, 3, and 4 in ascending order^22^. Common to the western and eastern zones, the four Pleistocene pyroclastic flow deposits and the clay, sand, and gravel layers interbedded between successive pairs of the pyroclastic deposits are distributed mainly within the 100-m-depth range and represent a local geologic feature. Post-glacial deposits (Shimabara Bay and Ariake clay layers) and the andesitic Togawa lava, with porous upper and lower portions and fractures in the intact portions, are distributed in the western and eastern zones, respectively^23^. The Togawa lava (Fig. 1b), which is situated stratigraphically between Aso-1 and Aso-2 and dated at 150 ka^22^, forms the major aquifer owing to its high permeability relative to that of the four pyroclastic flow deposits^24^. The hydrogeologic basement within the area is regarded as pre-Aso volcanic rock (Fig. 1c); however, the upper portion is locally well fractured and highly permeable, which forms a deeper aquifer^23^.

To preserve both the quality and the quantity of groundwater resources, continuous monitoring of groundwater levels has been implemented in 33 observation wells at 20 sites^25^. Among them, groundwater level data of 17 wells at 13 sites distributed within the study area (Figs. 1a and 3; the locations of these sites are marked with white circles enclosing the site numbers) were selected for this study. Most of the groundwater level data are freely available online. The depth range of the strainer section for pumping groundwater from the 13 wells and the lithology of the strainer section are summarised in Table 1. Two wells with different length and depth range of the strainer section for shallow and deep groundwater have been installed at four sites. The main lithologies of the strainer sections are classified as Togawa lava (4 sites: P1, P15, P16, and P33), Aso-1 (2 sites: P4 and P18), Aso-2 (5 sites: P3, P4, P9, P18, and P23), Aso-3 (4 sites: P7, P9, P24, and P32), Aso-4 (1 site: P15), Shimabara Bay layer (4 sites: P2, P6, P10, and P22) and pre-Aso volcanic rocks (1 site: P30). Regarding the relationship between surface lithology and topography, 6 sites (P1, P15, P16, P18, P30, and P33) are distributed in terrace deposits, 1 site (P4) is in pyroclastic flow deposits, and the other sites (P2, P3, P6, P7, P9, P10, P24, and P32) are in lowland coastal plain deposits (Fig. 1a). One important feature is that the well sites on the terrace are distributed along active fault traces.

**Table 1:** Details of groundwater observation wells. Location and elevation details of the 13 sites for the 17 groundwater observation wells, together with length, depth range of strainer section, and lithology of each section. Note that two wells with different lengths were installed at four sites for shallow and deep groundwater. “a.s.l.” represents “above sea level.”

Well No.	Elevation (m a.s.l.)	Length (m)	Strainer section (m)	Lithology
P1	16.9	55.5	39.0–50.0	Togawa lava
P2	4.6	35.3	22.3–33.3	Shimabara Bay layer
P3	4.6	110	97.0–108.0	Aso-2
P4	77.0	90	60.0–90.0	Aso-1, Aso-2
P6	5.4	45.7	31.7–42.7	Shimabara Bay layer
P7	5.4	154.5	121.5–138.0	Aso-3
P9	3.3	111.8	95.3–106.3	Aso-2, Aso-3
P10	3.3	41.2	24.7–35.7	Shimabara Bay layer
P15	7	25	Shallower than 25	Aso-4
P16	15.2	65.4	39.5–55.4	Togawa lava
P18	75.2	110	71.4–93.5	Aso-1, Aso-2
P22	4.2	80	36.1–47.1	Shimabara Bay layer
P23	4.2	115.3	82.3–98.8	Aso-2
P24	1.5	145	112.0–145.0	Aso-3
P30	39.3	137	120.5–131.5	Pre-Aso volcanic rocks
P32	3.5	125	113.0–129.5	Aso-3
P33	10.1	55	44.0–55.0	Togawa lava

### Characterization of precursory water level changes

**Figure 2:**
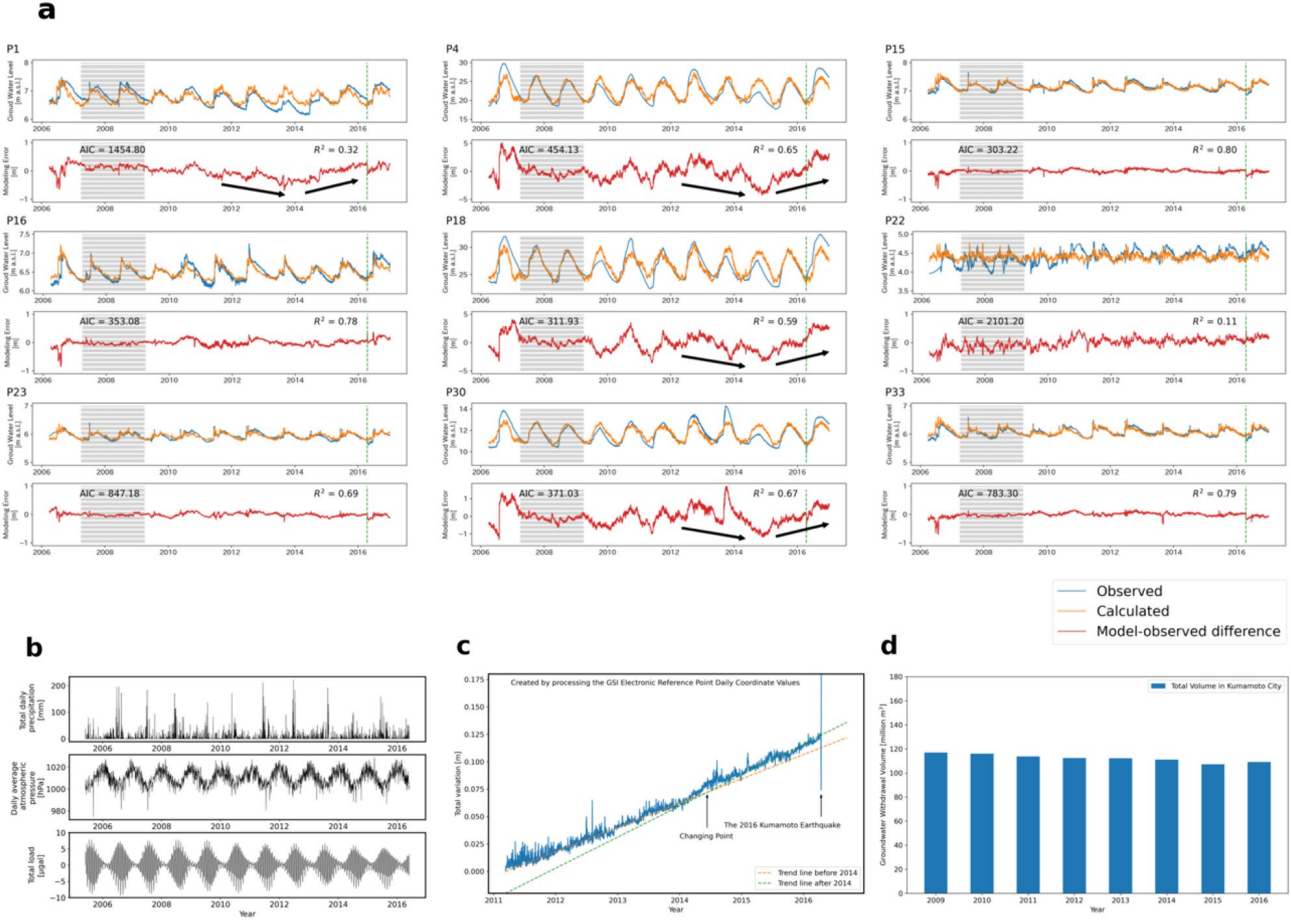
Comparison of observed and regression model-derived groundwater levels, meteorological data, GNSS station coordinate changes, and groundwater pumping amount. (**a**) Comparison between observed and regression model-derived groundwater levels (upper) with AIC and *R*^*2*^ values and residuals (model errors) at nine sites: P1, P4, P15, P16, P18, P22, P23, P30, and P33. Negative residual value means that the observed level is lower than the calculated level. The line at 2016 indicates the time of occurrence of the 2016 Kumamoto Earthquake and the portion shaded grey represents the training data period used for construction of the regression model. (**b**) Daily meteorological data comprising total precipitation and averaged atmospheric pressure observed at the Kumamoto district weather station (Fig. 1a), and theoretical daily mean displacement of the Earth surface attributable to the solid earth and ocean tides determined from GOTIC2 at site P1. (**c**) Temporal changes in the square root of differences in the three coordinates at the Kumamoto GEONET station (Fig. 1a) from the day after the occurrence of the 2011 Tohoku Earthquake. The target period was before the 2016 Kumamoto Earthquake. The changes can be approximated by two trend lines with different slopes. (**d**) Temporal change in the total amount of pumped groundwater in Kumamoto City^26^.

The groundwater level data acquired over 10 years were divided into two categories: training data and validation data, to avoid overfitting and to objectively detect the effect of the 2016 Kumamoto Earthquake on groundwater level change (Fig. 2a). The model training period extended from 1 April 2007 to 31 March 2009, i.e., approximately 2 years prior to the occurrence of the 2011 earthquake off the Pacific coast of Tohoku because the accuracy of groundwater level data increased from that time, owing to fewer missing values and absence of crustal deformation associated with large earthquakes. The distance between the study area and hypocentre of the Tohoku Earthquake is approximately 1300 km. The optimal temporal lag for the precipitation, atmospheric pressure, and earth tide displacement data (Fig. 2b) were specified by minimising the Akaike information criterion (AIC) for each observation well, and the coefficient of determination (*R*^*2*^) was calculated from the estimated and observed values of the model for the period from 1 April 2009 (the day after the training period) to 13 April 2016 (the day before the 2016 Kumamoto Earthquake), and its accuracy was evaluated for each observation well. The resulting coefficient of determination values confirmed that the predictive accuracy of the regression model using a 2-year training period was adequate.

Using the constructed optimal regression models based on the training period data, the groundwater levels were calculated by assigning the above three explanatory variables and comparing them with the observed levels at all the studied wells. The data for the eight wells with mostly minus residuals (model errors) indicate that the observed levels were lower than the calculated levels (Fig. 2a). Because the water level data in the lowland area, such as that at P22, does not show clear periodic changes and because the range of level change is small, the model-derived level does not agree well with the observed level. Consequently, the AIC and *R*^2^ values become large and small, respectively, expressing the low estimation accuracy. The other eight wells highlight conspicuous seasonal changes that can be followed by the three explanatory variables. The threshold for whether such seasonal changes are reproduced by the model is represented by an AIC value of 1500.

Among those models with AIC values of < 1500 (especially P15, P16, P23, and P33), the errors between the observed and model-calculated values are small, i.e., 5–10 cm, except for the section where the observed data were interpolated, and the *R*^2^ value is > 0.69. Although the AIC values of the other four sites (P1, P4, P18, and P30) are also < 1500, the errors are relatively large, i.e., 1–5 m. The residuals can be classified into one of two patterns: no trend close to zero over the target period at P15, P16, P23, and P33, and large fluctuation at P1, P4, P18, and P30.

Several factors must control the accuracy and applicability of the regression model other than precipitation, atmospheric pressure, and tidal displacement. Among them, the most plausible factors that were not included in the regression model as explanatory variables are the amount of pumped groundwater (as a typical anthropogenic factor) and the crustal strain of the regional stress field (as a natural factor). The amount of pumped groundwater in Kumamoto City, including in the study area, exhibits a monotonous linear decrease (Fig. 2d), according to an administrative report^25^. However, the scale of the reduction is small and the pumped amount does not show any seasonal or annual trends. Consequently, the effect of the pumped amount can be negated.

### Relationship between crustal strain and well strainer geology

The 17 wells were classified into three groups depending on the *R*^2^ value of the multivariate linear regression model accuracy: small error (*R*^2^ > 0.69), large error (0.69 > *R*^2^ ≥ 0.3), and inapplicability of the linear regression model (*R*^2^ < 0.3). Notably, the sites in each of these three groups are concentrated in areas with different topographic features: the small-error group is at the eastern edge of the lowland area (P15, P16, and P33 at three sites), the large-error group in the terrace area (P1, P4, P18, and P30 at four sites), and the inapplicable group in the lowland area (P2 & 3, P6 & 7, P9 & 10, P22, P24, and P32 at six sites).

**Figure 3:**
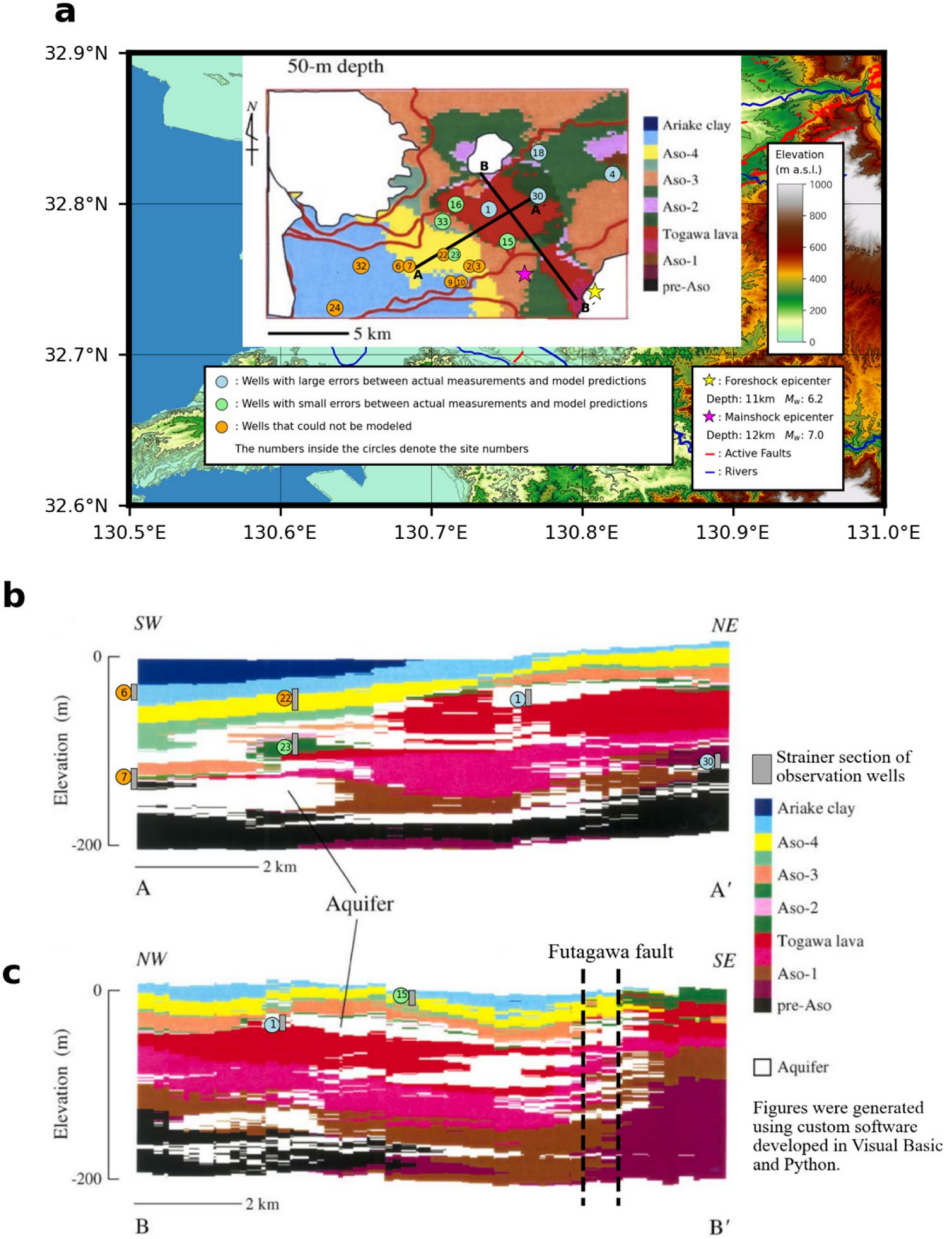
Horizontal and vertical cross sections of a two-dimensional geologic model showing the lithology of the strainer sections of the observation wells. (**a**) A horizontal slice of the geologic distribution at a depth of 50 m, (**b**) vertical slice of the geologic distribution along A–Aʹ, and (**c**) vertical slice of the geologic distribution along B–Bʹ from a three-dimensional geologic model using a geologic column dataset with estimated portions of aquifers (white portions)^24^.

The crustal strain effect is examined through consideration of the geologic structure and linear poroelasticity theory. Another noteworthy feature related to the geologic structure is that the lithology at the strainer sections of the four observation wells in the large-error group is weathered Togawa lava or the upper fractured portion of the pre-Aso volcanic rocks. The geologic distribution at the depth of 50 m, representing a horizontal slice of a three-dimensional geologic model constructed by a combination of spline and stochastic methods using a geologic column dataset^24^ (Fig. 3a), highlights the extensive distribution of the Togawa lava (Fig. 1b). By overlapping the wells, this map reveals that the small-error group is situated at the western boundary of the Togawa lava, and that P1 and P30 in the large-error group are in the Togawa lava and upper pre-Aso volcanic rocks, respectively (Fig. 1c). The lava distribution deepens towards the east and the depths of the strainer sections at P4 and P18. Another important observation derived from consideration of P1 and P30 is that the aquifer in the Togawa lava probably extends to the Futagawa fault, whereas the aquifer in the upper pre-Aso volcanic rocks is locally distributed (Fig. 3b,c).

The Togawa lava and upper pre-Aso volcanic rocks must be sensitive to such changes because of their high porosity and pore connectivity. Therefore, the source of the large model error in the wells at sites P1, P4, P18, and P30 can be attributed to the change in pore water pressure that can be expressed only by the crustal strain, not by the other three considered exploratory variables. As proof of this interpretation, the model errors were small for the four wells until the occurrence of the 2011 Tohoku Earthquake. However, after the earthquake event, the errors became large and remained so until mid-2014, when they reached the maximum value, decreased toward the occurrence of the 2016 Kumamoto Earthquake, and then returned to zero close to the time of the actual earthquake (Fig. 2a). The errors were all negative, meaning that the observed levels were lower than the model-calculated levels, probably because of the stress release after the Tohoku Earthquake.

The temporal change in crustal strain can be investigated using the *XYZ* coordinate data of each electronic reference point in the geocentric coordinate system, obtained from the F5 solution analysis of GEONET's data^27^. The *X*, *Y*, and *Z* axes are directed towards north, east, and vertically downward, respectively. The Geospatial Information Authority of Japan (GSI), which manages GEONET, has evaluated the RMS of residuals from regression curves considering linear trends, annual cycles, and semi-annual cycles as 2.56 mm in the *X* direction, 2.79 mm in the *Y* direction, and 7.03 mm in the *Z* direction^27^. The *XYZ* coordinates at the Kumamoto station (code: 950465) of GEONET (Fig. 1a) on 12 March 2011, the day after the Tohoku Earthquake, were fixed as the reference point, and daily differences from this reference ($dX$, $dY$, $dZ$) along each axis after that date were calculated. Then, the square root of the sum of the three components $S=\sqrt{{dX}^{2}+{dY}^{2}{+dZ}^{2}}$ was determined (Fig. 2c). The derived value *S* can be regarded as corresponding to the total amount of crustal strain at the station. Although *S* increased in a monotonic linear manner, this increase can be approximated by two trend lines with different slope: a relatively gentle slope before mid-2014 and a steeper slope subsequently. The range of the stability value of the F5 solution analysis of GEONET’s data becomes smaller than 0.01 m by transforming them into the *S* value. This *S* value < 0.01 m is much smaller than the magnitude of the trend change.

Notably, the time of change in the trend is broadly consistent with the time of change in the model error pattern (from increase to decrease) common to the four wells (Fig. 2a). The change from a gentle to steeper trend line suggests an increase in the crustal strain as a precursory phenomenon of the 2016 Kumamoto Earthquake. Therefore, the former pattern of increase in error can be attributed to stress release from the accumulated strain caused by the 2011 Tohoku Earthquake, which also suggests that the accumulated strain in the Togawa lava and the upper pre-Aso volcanic rocks was large and that it was released over a long period. Consequently, the observed groundwater levels became increasingly lower in comparison with the model-derived groundwater levels that did not consider the crustal strain effect. Meanwhile, the latter pattern of decrease in error might reflect an increase in compressive crustal strain towards the 2016 Kumamoto Earthquake.

Consequently, the groundwater level in a porous aquifer stratum such as the Togawa lava and the upper pre-Aso volcanic rocks can be considered an effective indicator of the change in crustal strain that occurs before and after the occurrence of a large earthquake. The change from decrease to increase at P1 occurred approximately one year earlier than at the other three sites (P4, P18, and P30). A possible reason for this phenomenon is that only P1 is in the aquifer connected to the Futagawa fault, which might indicate that the groundwater level in that region is more sensitive to change in crustal strain.

### Water level changes related to post-earthquakes

**Figure 4:**
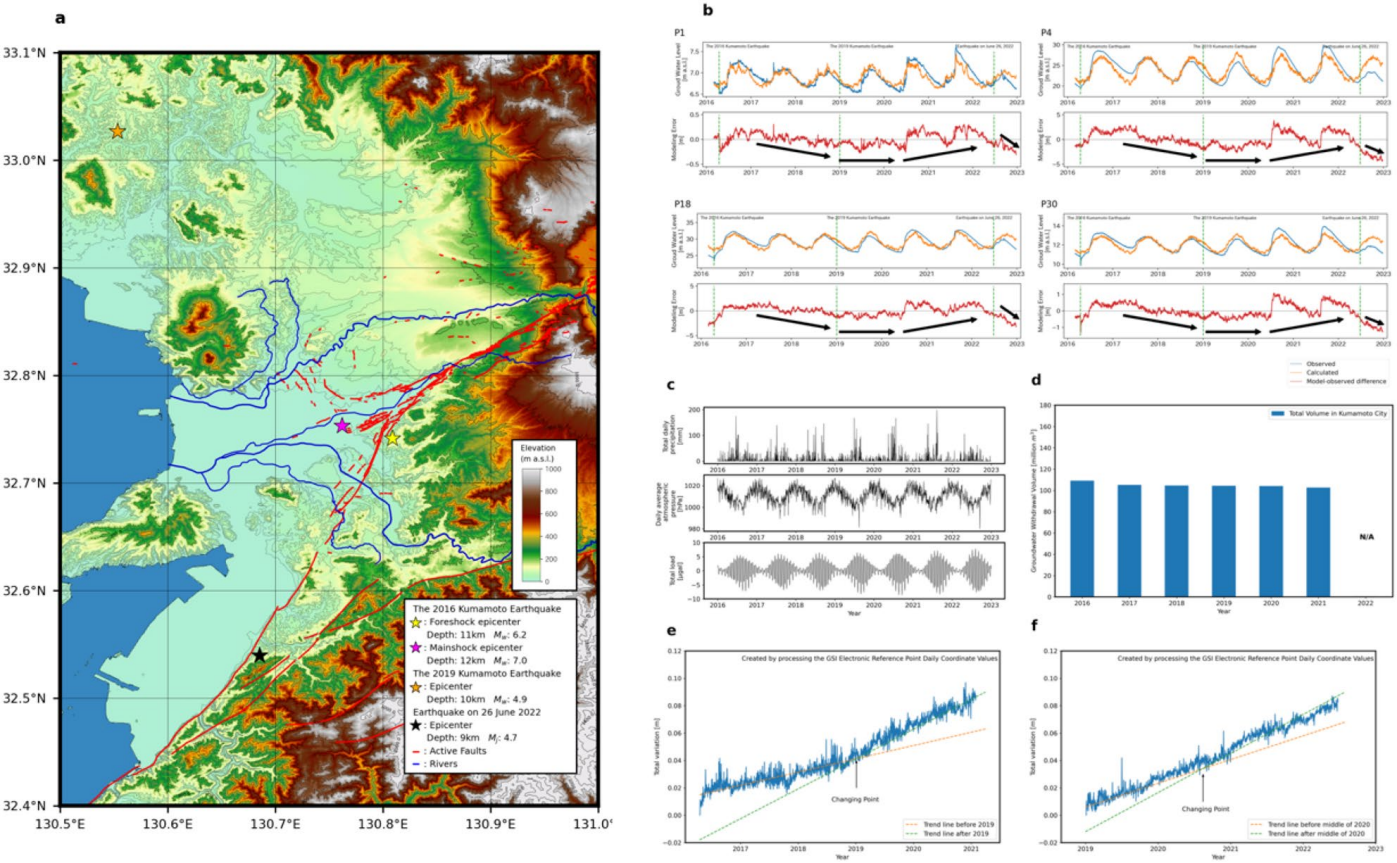
Comparison of observed and regression model-derived groundwater levels, meteorological data, GNSS station coordinate changes, and groundwater pumping amount. (**a**) Epicenters of the foreshock and the mainshock of the 2016 Kumamoto Earthquake and the earthquakes on 3 January 2019 and 26 June 2022, overlain with the topography and active fault traces in Fig. 1. (**b**) Comparison between the observed and regression-model-derived groundwater levels (upper) and residuals (model errors) at P1, P4, P18, and P30. A positive residual value means that the observed level is higher than the calculated level. The 2019 line represents the occurrence of an M_w_ 4.9 earthquake, and the 2022 line represents the occurrence of an M_j_ 4.7 earthquake. (**c**) Daily meteorological data comprising total precipitation and averaged atmospheric pressure observed at the Kumamoto district weather station (Fig. 1a), and theoretical daily mean displacement of the Earth surface attributable to the solid earth and ocean tides determined from GOTIC2 at site P1. (**d**) Temporal change in the total amount of pumped groundwater in Kumamoto City^26^. (**e**) Temporal change in the square root of the differences in the three coordinates at the Kumamoto GEONET station (Fig. 1a) from the day after the 2016 Kumamoto Earthquake and (**f**) the 2019 Earthquake. Common to the two periods, the changes can be approximated by two trend lines with different slopes.

Numerous aftershocks have occurred since the 2016 Kumamoto Earthquake, but the number of aftershocks has decreased and the seismic activity has calmed. A relatively large earthquake (Mw 4.9) occurred on 3 January 2019, with its epicenter (33° 1.6ʹ N, 130° 33.2ʹ E) at the depth of 10 km, i.e., approximately 36 km northwest of the epicenter of the 2016 Kumamoto Earthquake^15^ (Fig. 4a). A second earthquake occurred on 26 June 2022, with its epicenter (32° 31.8ʹ N, 130° 37.2ʹ E) at the depth of 9 km, i.e., approximately 28 km southwest of that^16^. The *S* value was calculated for these subsequent earthquakes by setting the *XYZ* coordinates on 18 April 2016 as the reference point (Fig. 4e). As before the 2016 Kumamoto Earthquake, the *S* value increased almost linearly, but again can be approximated by two trend lines with gentle and steeper slopes before and after the 2019 Kumamoto earthquake, respectively. This feature suggests that the trend in crustal strain magnitude around the Kumamoto area was changed by the earthquake, i.e., release from the effect of the 2016 Kumamoto Earthquake versus accumulation of compressive strain towards a subsequent earthquake. After the 2019 Kumamoto earthquake, S values were calculated by setting the *XYZ* coordinates on 5 January 2019 as the reference point (Fig. 4f). As in other analyses, the S value has been rising nearly linearly. However, around the middle of 2020, there was a shift in its slope. The trends before and after this point can be approximated by two trend lines, representing a gentle and a steeper inclination, respectively. This feature suggests that the trend in crustal strain magnitude around the Kumamoto area release from the effect of the 2019 Kumamoto Earthquake versus accumulation of compressive strain towards a subsequent earthquake.

Comparison of the errors between the model and the trend of the *S* value was conducted for the four wells of the large-error group (P1, P4, P18, and P30) (Fig. 4). In contrast to the situation before the 2016 Kumamoto Earthquake, the values of the model error gradually decreased until early 2019, with the error being minimal and negative. After the 2019 earthquake, the error increased in the positive direction and reached a maximum, followed by sharp decrease to negative values after the 2022 earthquake. Although the temporal trend in the error is less clear than before the 2016 earthquake, the timing of the change from decrease to increase is broadly consistent with the occurrence of the 2019 earthquake and the time of the change in the trend of the *S* value. Positive error can be interpreted as increase in the groundwater level attributable to pressure from crustal distortion, whereas negative error can be interpreted as an indication of reduction in pressure levels relative to when the model was created. The 2022 earthquake occurred at a time when the error was close to zero, as was the case for the 2016 earthquake. These results support the above interpretation that the observed groundwater levels that are markedly different from those calculated by the regression model using the meteorological and tidal factors are affected by the expansion/contraction of crustal strain.

## Discussion

The objective of this study was to detect precursory change in groundwater levels before the 2016 Kumamoto Earthquake through adoption of a multivariate regression model using precipitation, atmospheric pressure, and earth tide displacement data for the long term (10 years) in 17 observation wells at 13 sites in the Kumamoto area. The optimal regression models were determined through AIC. The 17 wells were classified into the three groups based on the fitting degree to the regression models: small error, large error, and inapplicability of the linear model. The three groups showed a clear distributional feature, i.e., the small-error group was concentrated at the boundary between the terrace and lowland areas, the large-error group was concentrated in the terrace area, and the inapplicable group was concentrated in the lowland area. The large-error group comprising four wells was considered the most important because the porous Togawa lava and the upper fractured pre-Aso volcanic rocks were the lithologies at the strainer sections, and the negative model errors revealed a distinct trend of increase until mid-2014, followed by a decrease towards the 2016 Kumamoto Earthquake. Therefore, the observed levels were consistently lower than the model-derived levels. This trend and the timing of the change in the trend broadly matched the temporal change in the crustal strain that was interpreted from the change in the *XYZ* coordinates at the GNSS station in the study area. The increase and decrease in the trend of the errors were most likely caused by the release of the accumulated compressive strain associated with the 2011 Tohoku Earthquake until mid-2014 and the gradual increase in the compressive strain in the Togawa lava and the upper pre-Aso volcanic rocks that finally linked to the 2016 Kumamoto Earthquake.

This interpretation of the crustal-strain dependence of the groundwater table was validated through case studies of two earthquakes that occurred in 2019 and 2022. Because these earthquakes were much smaller than the 2016 earthquake, changes in the model errors were not evident. Nevertheless, the changes in the groundwater level in the Togawa lava and the upper part of the pre-Aso volcanic rocks seemed to reflect the effect of strain release from the 2016 earthquake before the 2019 earthquake, increased compressive strain after the 2019 earthquake, and strain release from the 2022 earthquake. The findings of this study reveal that porous aquifer strata are sensitive to crustal strain, and that changes in the groundwater level in such aquifers must be an effective indicator of the expansion/contraction of crustal strain.

## Methods

### Multivariate analytical model for estimating groundwater levels

As a pre-processing step, all variables were normalised to the mean 0 and variance 1. The normalised groundwater level $gwl_{t}$ at a given site and time $t$ (*t* = 1, 2… *n*: where *n* is the number of data used to construct the model) was correlated with the explanatory variables of precipitation $r_{t}$, atmospheric pressure $p_{t}$, and earth surface displacement by tidal forces $e_{t}$ using the multivariate linear regression model expressed as follows:

1 $$g w l_t=\sum_{i=0}^{m_1} \alpha_i r_{t-i}+\sum_{j=0}^{m_2} \beta_j p_{t-j}+\sum_{k=0}^{m_3} \gamma_k e_{t-k}+\varepsilon_t$$

where $\varepsilon _{t}$ is the white-noise residual following a normal distribution with mean 0 and variance $\sigma ^{2}$; $m_{1}$, $m_{2}$, and $m_{3}$ are the model regression order; and $\alpha$, $\beta$, and $\gamma$ are regression coefficients. The optimal order of ${m}_{1}$, ${m}_{2}$, and ${m}_{3}$ is determined using the Akaike information criterion (AIC) formulated as follows:

2 $$AIC=-2 \ln L+2 k$$

where *L* is the likelihood of the regression model and *k* is the number of free parameters. In this study, *k* = $m_{1}+ m_{2}+m_{3}+4$. The model that minimises the AIC is regarded as the best model that can balance most adequately the smallness of the model error and the complexity of the model while avoiding overfitting.

A logarithm of *L* ($\ln L$) is generally used for model determination:

3 $$\ln L= {-n/2}{\log 2}{\pi\sigma^2} - \frac{1}{2\sigma^2}\sum_{t=1}^n (gwl_t-\sum_{i=0}^{m_{1}}\alpha_{i}r_{t-i}-\sum_{j=0}^{m_{2}}\beta_{j}p_{t-j}-\sum_{k=0}^{m_{3}}\gamma_{k}r_{t-k}$$

The maximum likelihood estimate of the variance ${\widehat{\sigma }}^{2}$ is then defined as follows:

4 $$\hat{\sigma}^2=\frac{1}{n} \sum_{t=1}^n\left(g w l_t-\sum_{i=0}^{m_1} \alpha_i r_{t-i}-\sum_{j=0}^{m_2} \beta_j p_{t-j}-\sum_{k=0}^{m_3} \gamma_k e_{t-k}\right)^2$$

By substituting Eq. (4) in Eq. (3), the maximum $\ln L$ is defined as follows:

5 $$-\frac{n}{2} \log 2 \pi \sigma^2-\frac{n}{2}=\frac{n}{2}(2 \pi+1)-\log \hat{\sigma}^2$$

Accordingly, maximisation of $\ln L$ is equivalent to maximisation of ${\widehat{\sigma }}^{2}$, which is equivalent to a problem of the usual least squares method. Because ${\widehat{\sigma }}^{2}$ is a function of ${m}_{1}$, ${m}_{2}$, and ${m}_{3}$, the AIC can be rewritten as follows:

6 $$AIC\left(m_1, m_2, m_3\right)=\frac{n}{2}(2 \pi+1)-n \log \hat{\sigma}^2\left(m_1, m_2, m_3\right)+2\left(m_1+m_2+m_3+4\right)$$

The optimal values of $m_{1}$, $m_{2}$, and $m_{3}$ can also be determined by minimising the AIC.

### Theoretical value of earth’s surface displacement due to tidal forces

Despite representing a smaller effect than that of meteorological factors (precipitation and atmospheric pressure), earth’s tidal forces also change groundwater level^28^. There are two tidal forces: the solid earth tide caused by gravitational action mainly associated with the sun and moon, and the ocean tide caused by elastic deformation of the crust attributable to change in the loading of seawater on the seafloor. The displacement and strain at the earth’s surface by these two tides were calculated theoretically using the Fortran code GOTIC2^29^ that uses a convolution of the ocean tidal load distribution and Green’s function. The coordinates of each groundwater level observation well and the elevation of the pipe head were entered into GOTIC2 to calculate the hourly theoretical displacements. The calculated hourly theoretical displacements were transformed into daily mean displacements, for consistency with the groundwater level data and the meteorological data, and then used as an explanatory variable.

## Data Availability

All the groundwater level observation data were obtained from the website of the Water Conservation Division of the Environmental Promotion Department of the Kumamoto City Environment Bureau (https://www.city.kumamoto.jp/kankyo/), with periods of missing data in the online records provided as an additional supplement. All meteorological data necessary to support the results of this study, including daily changes in precipitation and mean atmospheric pressure at the Kumamoto district weather station (32° 48.8ʹ N, 130° 42.4ʹ E, 37.7 m a.s.l.), are available on the website of the Japan Meteorological Agency (https://www.data.jma.go.jp/obd/stats/etrn/). All data of “daily coordinate values” at the Kumamoto observation point (No. 950465, 32° 50.2ʹ N, 130° 45.4ʹ E) from the GNSS continuous observation system (GEONET) of the Geospatial Information Authority of Japan are available for download (https://terras.gsi.go.jp/pos_main.php).

## Code availability

The codes used to generate and analyse the data presented in this paper are available from the corresponding author upon reasonable request.
